# Best Practices for Virtual Engagement of Patient-Centered Outcomes Research Teams During and After the COVID-19 Pandemic: Qualitative Study

**DOI:** 10.2196/24966

**Published:** 2021-03-11

**Authors:** Erin K Thayer, Molly Pam, Morhaf Al Achkar, Laura Mentch, Georgia Brown, Traci M Kazmerski, Emily Godfrey

**Affiliations:** 1 Department of Family Medicine University of Washington Seattle, WA United States; 2 Cystic Fibrosis Reproductive and Sexual Health Collaborative Seattle, WA United States; 3 Department of Pediatrics University of Pittsburgh Pittsburgh, PA United States

**Keywords:** attributes, best practices, COVID-19, cystic fibrosis, engagement, outcome, patient, patient-centered outcomes research, qualitative, research, stakeholder engagement, user guide, virtual care, virtual teams, web-based collaboration

## Abstract

**Background:**

Patient-centered outcomes research (PCOR) engages patients as partners in research and focuses on questions and outcomes that are important to patients. The COVID-19 pandemic has forced PCOR teams to engage through web-based platforms rather than in person. Similarly, virtual engagement is the only safe alternative for members of the cystic fibrosis (CF) community, who spend their lives following strict infection control guidelines and are already restricted from in-person interactions. In the absence of universal best practices, the CF community has developed its own guidelines to help PCOR teams engage through web-based platforms.

**Objective:**

This study aimed to identify the important attributes, facilitators, and barriers to teams when selecting web-based platforms.

**Methods:**

We conducted semistructured interviews with CF community members, nonprofit stakeholders, and researchers to obtain information regarding their experience with using web-based platforms, including the effectiveness and efficiency of these platforms and their satisfaction with and confidence while using each platform. Interviews conducted via Zoom were audio recorded and transcribed. We identified key themes through content analysis with an iterative, inductive, and deductive coding process.

**Results:**

In total, 15 participants reported using web-based platforms for meetings, project management, document sharing, scheduling, and communication. When selecting web-based platforms, participants valued their accessibility, ease of use, and integration with other platforms. Participants speculated that successful web-based collaboration involved platforms that emulate in-person interactions, recognized the digital literacy levels of the team members, intentionally aligned platforms with collaboration goals, and achieved team member buy-in to adopt new platforms.

**Conclusions:**

Successful web-based engagement in PCOR requires the use of multiple platforms in order to fully meet the asynchronous or synchronous goals of the project. This study identified the key attributes for the successful practice of PCOR on web-based platforms and the common challenges and solutions associated with their use. Our findings provide the best practices for selecting platforms and the lessons learned through web-based PCOR collaborations.

## Introduction

Patient-centered outcomes research (PCOR) entails patients and stakeholders partnering with researchers to define research questions, design studies, interpret findings, and generate schema to disseminate information among patients and communities [1]. Authentic collaboration among researchers, providers, and community members requires open lines of communication and trust [2]. During the COVID-19 pandemic, PCOR teams must consider not only the management of team dynamics but also the technology they would use to facilitate successful collaboration. PCOR teams have acknowledged the need to rapidly adapt to web-based team interactions; hence, the demand for web-based operating guidelines has increased [3-6]. While PCOR is traditionally conducted in person, social distancing is now recommended in most cities and states during the COVID-19 pandemic, forcing PCOR teams to collaborate on web-based platforms for continued engagement.

Studies examining virtual team science emphasize the need for web-based technology for meetings, scheduling, day-to-day correspondence, task management, and document sharing, among other purposes [2,7]. The shift from in-person interactions to web-based interactions appears simple; however, evidence indicates that web-based collaboration requires more attention to team dynamics, as conflict and problems in coordination may arise [8]. Additionally, building strong interpersonal connections and trust among team members can be more challenging in a remote working environment [8]. When some team members are colocated and others are geographically dispersed, certain in-groups and out-groups might be unintentionally formed, which can lead to tension and feelings of exclusion among some group members [9].

Our PCOR team, composed of adults with cystic fibrosis (CF), academic researchers, and staff, has only interacted remotely since its establishment in 2016. CF is a rare, multisystem progressive genetic disease. One of its hallmarks is the high risk of persistent lung infections, which causes permanent damage. These infections render individuals with CF at a high risk of cross-infection [10,11]. In 2003, the Cystic Fibrosis Foundation established infection control guidelines to reduce the risk of cross-infection among individuals with CF [12]. Updated in 2013, the guidelines now suggest that individuals with CF should always practice social distancing, staying 6 feet apart from other individuals with CF [12]. Therefore, to support interpersonal connections, the CF community has developed an extensive web-based community, including support groups and medical or scientific conferences [13,14]. The CF community has thus provided a wealth of guidelines for web-based engagement, particularly during the COVID-19 pandemic.

Our study seeks to guide PCOR teams transitioning to web-based community engagement in selecting the best web-based platforms to sustain authentic interactions among all team members.

## Methods

### Study Design

We conducted qualitative semistructured interviews within an interpretivist paradigm, in which researchers and research participants develop interpretive frameworks to design questions and corresponding responses [15].

Because of its long-standing experience with web-based engagement, the CF community constitutes a primary stakeholder. We interviewed individuals with CF, caregivers of individuals with CF, and employees of a CF advocacy organization. We also interviewed researchers, research staff, and several employees at a training institution. The University of Washington Institutional Review Board approved this study (IRB 6146). Three patient partners (GB, LM, and MP) participated as team members and were engaged throughout the study.

### Participant Recruitment

We used purposive and snowball sampling to target individuals for PCOR, who collaborated mostly or solely through web-based platforms [16]. We aimed to enroll enough participants to reach saturation [17]. Because few PCOR teams engaged only through web-based platforms during recruitment, we expanded our eligibility criteria to include any research team member who self-identified as collaborating either mostly or solely through web-based platforms. Within the CF community, we recruited participants through our partner organizations including the Cystic Fibrosis Reproductive and Sexual Health Collaborative; Cystic Fibrosis Foundation; Cystic Fibrosis Research, Inc; and their networks. Outside the CF community, we recruited participants through the North American Primary Care Research Group’s Patient and Clinical Engagement Program, the American Academy of Family Physicians, the National Patient-Centered Clinical Research Network, and the University of Washington’s International Training and Education Center for Health. Participants were categorized by stakeholder groups defined by the PCOR Institute [18]. Under these definitions, “patients” include both patients with CF, their caregivers, and advocates; “researchers” include researchers and research staff; and “training institutions” include those that deliver education on health professions or represent the organizations that provide such programs.

### Data Collection

We developed our semistructured interview guide on the basis of 3 components of usability: effectiveness, efficiency, and satisfaction [19,20]. Brooke [20] defined *effectiveness* as the ability of users to complete tasks and achieve goals, *efficiency* as the extent to which users expended resources to achieve their goals, and *satisfaction* as the level of comfort users experience while achieving their goals. We asked participants what web-based platforms their teams or collaborators use, their experience with these platforms, and their perceptions of the effectiveness, efficiency, and satisfaction with each platform. Multimedia Appendix 1 displays our interview guide.

The interviewer (EKT) was experienced with qualitative research methods. She is a White, cisgender graduate student at the University of Washington and was blinded to the identities of the participants prior to the interviews. On providing informed consent, the study participants were interviewed remotely through Zoom videoconferencing, and the audio in the meetings was recorded. Participants were offered a gift card for up to 45 minutes of their time. At least one other team member transcribed and reviewed each interview for accuracy.

### Data Analysis

We performed content analysis as described by Elo and Kyngäs [21]. We developed a codebook based on 3 robust interviews with inductive and deductive coding approaches [22,23] using the a priori domains “ease of use,” “efficiency,” and “satisfaction.” On developing the codebook, we used team-based coding [22]. Two independent research team members (EKT and MP) coded all interviews using Dedoose qualitative analysis software [24]. When discrepancies occurred, excerpts were read again to clarify the meaning of the code and the selected text. EKT led the group to review codes, resolve discrepancies to obtain consensus, and develop the themes through an iterative process [22].

Furthermore, we used PCOR Institute’s 6 principles of engagement (reciprocal relationships, colearning, partnerships, transparency, honesty, and trust) [1] to guide the reconstruction of the themes. We redeveloped and reorganized themes to make our findings practical and usable as guidelines, and this process helped us identify key considerations and challenges for PCOR teams collaborating through web-based platforms.

## Results

We interviewed 15 participants belonging to three separate stakeholder groups—patients, researchers, and teaching institutions (Table 1)—between January and February 2019. Each interview was conducted on a one-on-one basis, although one interview included two interviewees. In total, 10 participants had team members based solely in the United States, and 5 had members outside the United States. Furthermore, we reviewed the participant considerations in the selection of web-based platforms and the common challenges participants faced while collaborating through web-based platforms, along with proposed solutions for these challenges.

**Table 1:** Participant characteristics (N=15).

Stakeholder group	Stakeholders, n (%)
Patients	4 (26.7)
Researchers	6 (40.0)
Training institutions	5 (33.3)

### Technological Considerations

Respondents noted that every team member needs to have the proper technology to be equal contributors to research discussions. This is especially important for adhering to the PCOR principles of transparency, partnership, and colearning. We included the following 5 considerations. 

#### Variability in Internet Connections

To ensure team cohesion and good communication, participants with either low-bandwidth internet connections or no internet access need accommodations or alternatives to connect and engage with others. Respondents reported that certain videoconferencing platforms were better equipped to handle low-bandwidth internet connections than others. Teams should avoid platforms that deliver inconsistent services, which can lead to poor video quality and cause computers to crash.

We were having more audio issues with [video conferencing platform] within our country offices. But they were greatly reduced once we started using [a different video conferencing platform].

Participant #6; training institution

#### Availability of the Necessary Technology

Respondents noted that every team member needs to have access to a camera and speaker system, which is doable with platforms that operate on different devices, such as computers, cellphones, or tablets. Respondents cited equipment disparities as a barrier to successful web-based collaboration.

It would be very important for people who are regularly using online meeting[s] to get the webcam and speaker system just because it… streamlines everything so much. And also, I think the face-to-face is really nice, but most people seem to not have webcams.

Participant #8; researcher

#### Institutional Firewalls

Every PCOR team member should be able to easily log into the platforms to effectively engage with others. Participants cited onerous logins and restricted access or institutional firewalls as a barrier to communication and collaboration among teams with community members or patients.

One thing I don’t like about [document sharing platform] is that it is not possible for me to give access to someone outside [the university]. So, if I’m working on a project where I’m collaborating with someone at another institution or in a community setting, it's really hard to get them access to a file that’s related to a project. So that is one disadvantage of having [institutional access].

Participant #15; researcher

Even certain platforms that do not require an institutional login have requirements that hinder easy access.

[Document sharing platform] is good, except for the fact that you have to have a [specific email] address. So, we’ve worked with some people that need access to [a project] but they don’t have a [specific] address… So, they had to create a separate email address and have a password to access it.

Participant #10; researcher

#### Accommodation for Multiple Languages

For PCOR teams engaging members who are not fluent in English, participants highly rated platforms that offered translation services.

I stumbled onto the translation function available in that version of [video conferencing platform], which was great, because one of our managers is in Mozambique. She speaks Portuguese and her English is proficient, but there are times that we struggle in our communication… It allowed us to type in our native language and then translate it into the recipient’s language.

Participant #6; training institution

#### Cost

Cost was an especially important consideration for teams with collaborators who have fewer financial resources. Most participants preferred platforms that were either sponsored at low or no cost by an institution or those with free public access.

In terms of meetings, [video conferencing platform] has been really good, because everyone has access to it and it's mostly free. So that is good in terms of equity for us and our country partners or partners in other resource limited countries.

Participant #13; training institution

Multimedia Appendix 2 summarizes the attributes of various tools in web-based platforms for engagement, which were noted by participants interviewed in this study. Considering the breadth of platforms available to teams, we have provided additional details regarding the attributes valued by participants, such as security and privacy, along with other noteworthy benefits and challenges.

### Challenges and Solutions for Successful Engagement on Web-Based Platforms

Participants voiced several challenges associated with successful engagement on web-based platforms. We grouped these challenges into 4 separate themes and indicated participants’ solutions for each challenge.

#### Aligning Platform Selection With Collaboration Goals

##### Challenge

One challenge was the misalignment between the tools in web-based platforms and type of communication. For example, short, quick messages delivered through instant messaging (or texting) appeared to lack nuance and were often misinterpreted, especially if the communication required refinement and explanation.

When you’re writing an email you elaborate, but when you’re using [instant messaging], sometimes you have a few sentences or a few words and it might be perceived differently than the message you wanted to send.

Participant #13; training institution

##### Potential Solutions

Participants emphasized a need for groups to establish policies regarding the choice of platform and the intended purpose (eg, email vs instant messaging vs text messaging). For videoconferencing, respondents indicated that their teams followed certain rules when using the chat feature to reduce cross-talk, which still allow participants to comment (in written form) in real time or appoint a videoconference leader to facilitate the discussion and monitor the conversation for any cross-talk or feedback, muting team members whenever necessary.

..As we’ve built out our community engagement efforts, we’ve actually created best practices for other teams who are collaborating with community members on how to host a virtual meeting in the best possible way.

Participant #3; patient

#### Resembling In-Person Interactions

##### Challenge

Participants reported that interactions on web-based platforms are not the same as those in person because of the loss of nuances that commonly occur during face-to-face interactions. Additionally, participants indicated that it was difficult to develop personal connections with other team members when collaborating on web-based platforms.

##### Potential Solutions

Videoconferencing and instant messaging platforms resemble in-person interactions by providing the following advantages: (1) facilitation of verbal and nonverbal communication, (2) focus and accountability, and (3) instant connectivity.

###### Verbal and Nonverbal Communication

Participants appreciated videoconferencing platforms because they allowed them to simultaneously see facial expressions and body language while other team members spoke, which facilitated deeper understanding and collaboration.

It’s neat to see, especially with the video, how connected I can feel to people who are working across the country. I see these faces every month, hear these voices, but [when] you can see their face, it feels more connected.

Participant #8; researcher

Another participant concurred with the importance of video for engagement.

Having the ability to ...connect via video chat has changed the way we work with the community… If we were still having phone line conference calls, it would be a disservice to the engagement work that we do. It’s as close to face-to-face as we can do.

Participant #3; patient

Other participants believed that the use of videoconferencing platforms helped team members track the conversation and navigate awkward cross-talk because of the ability to see body language.

###### Focus and Accountability

Participants indicated that video also helped ensure that others paid attention to the conversation. Several participants described how videoconferencing added a level of focus or accountability similar to that during in-person meetings.

Because the camera is on, you’re accountable. You have to pay full attention to meetings, so that’s been great.

Participant #13; training institution

###### Instant Connectivity

Participants favorably described the instant connectivity associated with instant or text messaging. Participants speculated that instant messaging fostered greater cohesion when completing tasks and minimized work delays that often arise with regular email.

It’s just that instant connectivity. As opposed to waiting until the next day particularly with delays when you’re working globally. Now you are able to have that instantaneous communication, direct link, to one another.

Participant #6; training institution

Additionally, participants described instant messaging as resembling spontaneous, office-based “water cooler” conversations, which are potentially more social and personal in nature. This phenomenon was noted in a team with members based in Seattle (WA, United States) and Harare (Zimbabwe).

[We have] a [Instant Messaging Platform] group where we send each other little messages about some office things and a lot of times social things: holiday greetings or somebody’s baby was born.

Participant #2; training institution

#### Learning and Adopting the Technology

##### Challenge

Participants noted the challenges associated with the use of the technology among some participants because of a lack of digital literacy (ie, not being “tech savvy”) or needing extra time with new or frequent software updates.

This is a newer version of [video conferencing and instant messaging platform] and I wasn't able to find the translation. I just spent a couple minutes going, ‘I wonder where that is?’…and I realized ‘oh, I'd have to spend more time to dive deeper to find where that functionality is.’ I am aware that that functionality exists, but I don't know how to get to it.

Participant #6; training institution

Another challenge in this category, which participants cited, was achieving buy-in from team members to adopt a new web-based tool.

When something new comes out, it creates like ‘Well, why do I need to use a different program management tool, this program management tool is working just fine for me.

Participant #6; training institution

##### Potential Solutions

Designating a team technology champion as the “go-to” person to help select appropriate communication platforms and spend extra time assisting members with relatively lower digital literacy were noted as solutions to ensure every team member can learn and adopt the technology. Other solutions included selecting tools that are simple, intuitive, familiar, and quick to learn among team members or setting aside time during a meeting for all team members to learn the new platform.

When you aren’t comfortable with [a platform] you have to put more effort into it. Depending on the complexity of specific tasks in [the platform], people might be less comfortable using it...You have to take some time to learn the software.

Participant #13; training institution

Some participants found it easier to adopt current, mainstream platforms rather than new, customized platforms.

I think people’s familiarity with [frequently used platform] and the fact that many people are within that Google environment, just makes it a viable option.

Participant #6; training institution

Another solution suggested by participants was to generate buy-in for platform adoption by persuading an adequate number of team members to post important, interesting, or new content on the platform to entice reluctant adopters to access it.

You have to be constantly putting content out onto the [instant messaging] platform to keep people engaged or you run the risk of falling off and not checking it.

Participant #1; patient

Furthermore, participants suggested adhering to the platform long enough for it to become habitual for all team members, regardless of varying digital literacy levels.

It has nothing to do with a computer skill level or an intelligence level or competency. It is just simply the more you do it, the faster and easier it becomes.

Participant #14; patient

#### Improving Team Efficiency on Web-Based Platforms

##### Challenge

Participants reported that some platforms are inefficient (eg, the use of email for document editing), leading to multiple versions of the same document and reducing the goal of team efficiency and productivity. Furthermore, signing into multiple web-based platforms was a barrier to team efficiency and productivity.

##### Potential Solutions

Participants reported that videoconferencing chats, especially with another moderator’s assistance, and screen sharing features increased productivity. Other participants suggested that using a web-based platform for multiple individuals to edit a single document (eg, Google Drive and Egnyte) helped manage the versions of a document and minimized the need for additional discussions through email, videoconferencing, or instant messaging.

We can all be working on the same file and not have to email it and have 50 different versions floating around. This way we can have one version on [document sharing platform]. We know that’s the one we are working on, which is incredibly helpful.

Participant #11; researcher

Additionally, participants encouraged teams to select platforms that integrate with one another to reduce the burden of checking or signing into multiple platforms. For example, Google Drive and Slack (an instant messaging platform) integrate such that Google documents can be previewed, opened, shared, or saved in Slack; this prevents the need to switch to another platform (eg, email) to exchange documents. Platforms that integrate with computer desktops help avoid repeated downloading and reuploading of documents, facilitating easy access to the document among all team members.

[Document sharing platform] has office integration… So, we can basically work directly on the desktop and everything saves up to [the online platform], so nothing actually ever touches our local workstation.

Participant #11; researcher

## Discussion

### Principal Findings

This study identified platforms that emulate in-person interactions, such as videoconferencing or instant messaging platforms, which have helped regain nuances and social connections that are lost owing to the lack of in-person interactions, particularly during the COVID-19 pandemic. When selecting the appropriate platform tools to use, PCOR team members should consider the infrastructural requirements of the team for access to and comfort with individual platforms. These considerations, in turn, would facilitate the selection of web-based platforms and engagement strategies. Although this study does not provide an exhaustive list of platforms available, our findings would help streamline the selection of such platforms for teams by highlighting certain attributes considered to be of high value by our study participants, and consider other platform benefits and challenges when engaging solely through web-based platforms.

Concurrent with our findings, the National Research Council Committee on the Science of Team Science reported that the use of both video and screen sharing during videoconferencing helps ensure accountability and focus by visualizing facial expressions, body language, and directing attention using a mouse or pointer [2]. Moreover, the National Research Council reported that these nuances can be interpreted differently in different cultures and their implications can be misconstrued, thus emphasizing the need to establish guidelines for the use of the platform on initiating new collaborations [2]. While instant messaging does not emulate in-person interactions, it allowed teams to rapidly discuss work-related queries and provided space for social interaction. Similarly, other studies have reported that instant messaging is effective for brief work-related communications and discussions, and to maintain social interaction [25,26].

In addition to our findings regarding the importance of selecting platforms that are easy to use, accessible, compatible with other platforms, and of low or no cost, a previous study reported security and privacy, levels of control, and response speed as necessary considerations [27]. Further, we found that access to a reliable internet connection is an important consideration. One study reported that interruptions in video transmission resulting from technical difficulties can be highly disruptive to conversational flow and collaboration [28]. Reliability of an internet connection is potentially important during the COVID-19 pandemic because almost all team members work from home. Further, our study participants reported they stopped sharing video or switched to a telephone call or teleconference when the internet connection was inconsistent. Additionally, if team members speak different languages or require other accommodations, teams should consider platforms with a translation feature. Notably, none of our study participants required accommodation for disabilities; nonetheless, closed captioning and text-to-speech reader features are available on some platforms [29,30].

The National Research Council reported that if a platform is difficult to use, does not align with the team’s activities, or does not integrate well with other platforms, it would probably deter collaboration and team efficiency and eventually be abandoned [2]. Our study participants offered additional strategies to establish guidelines to use specific platforms and to designate a technology champion who can spend additional time with members who are less technologically savvy. Similarly, Berente and Howison [31] suggest that web-based collaborations are successful when they establish and maintain guidelines on the use of platforms, particularly when team members rotate across projects or work on multiple projects across different teams and institutions, as is common in PCOR and among other research teams. Since many platforms are designed for full-time use by teams, PCOR teams should consider patient partners, and other external stakeholders may benefit from adhering to one platform so that it becomes familiar to all members.

The unique feature of our study is that it examined methods to collaborate with community members on a research team through a web-based platform. While other studies have reported accessibility as an important attribute to consider when selecting web-based platforms, our study found that specific subthemes under accessibility were particularly important when engaging patient partners and community members. Additionally, Multimedia Appendix 2 provides an opportunity for teams to review attributes of common web-based platforms before implementing them onto the team.

### Limitations

Several limitations in our study warrant mention. We had intended to enroll only PCOR teams to make our findings more applicable to this population; however, we found that few PCOR teams were already engaging solely on web-based platforms when we conducted our interviews in 2019. The CF community was our key patient and advocacy stakeholder group because individuals with CF have a lifelong requirement to maintain social distance from other individuals with CF and have extensive experience with web-based collaboration. Although we enrolled other research and not-for-profit teams in this study, our findings may not be generalizable to nonresearch teams. Additionally, considering the dynamic nature of web-based platforms and software programs, we recognize that some of the platforms indicated in this study may stop being available. Although we included a diverse group of participants, including individuals with CF, researchers, other nonprofit stakeholders, their perspectives may not be generalizable to all the members of their group. Nonetheless, we believe that many of the valued attributes highlighted by our study participants would still hold true, even as new programs and tools enter the global market.

### Conclusion

This study provides valuable perspectives of PCOR and other research teams that engage through web-based platforms to establish guidelines for teams that were either already collaborating through such platforms or were forced to transition to such platforms owing to the COVID-19 pandemic. Our findings provide a roadmap for PCOR collaborations with considerations for selecting web-based platforms on the basis of individual team requirements, and solutions to potentially common challenges faced by research teams collaborating through web-based platforms. A guide for engagement on web-based platforms generated on the basis of our findings is available on the internet [32].

## Appendix

Multimedia Appendix 1 Semi-structured interview questions.

Multimedia Appendix 2 Benefits and Challenges of Specific Online Platforms.

## Abbreviations

CF: cystic fibrosis
PCOR: Patient-Centered Outcomes Research
