# Supplementary material for: Best Practices for Virtual Engagement of Patient-Centered Outcomes Research Teams During and After the COVID-19 Pandemic: Qualitative Study
Source: J Particip Med. 2021 Mar 11;13(1):e24966. doi: 10.2196/24966 (PMC7954110; doi:10.2196/24966)
Supplement: Multimedia Appendix 1 [file jopm_v13i1e24966_app1.docx]

## **Multimedia Appendix 1.** Interview Questions

1. What online platforms does your team or collaboration use?
2. Describe your experiences using this/these platforms.
   - Why were these platforms chosen? What were the goals/objectives of using this platform?
   - How well did these platforms perform in meeting your goals/objectives?
   - What are the advantages?
   - What are the disadvantages?
3. What kinds of tasks do you find easy to complete using this/these platforms?
   - Does this vary for other users?
4. What kinds of tasks are difficult for you to complete using these platforms?
   - How does this vary by user?
5. What are the specific tasks you hoped to perform in this platform?
   - Do these tasks differ by user?
   - If tasks differ for each user, please list them by user type
6. How would you rate your current confidence in performing tasks on these platforms?
   - How would you rate the confidence of other users performing various tasks on these platforms?
7. How much time did you and the other users spend learning these platforms before performing tasks?
8. Were there any issues that caused you to seek technical support while using these platforms?
   - What were they?
   - How easily were those issues resolved?
9. How many tasks are you able to complete using these platforms?
10. Do various tasks require more, or less time to complete than you initially expected?
11. Overall how would you rate your experience using these platforms?
    - How would other users rate their experience?
12. How often did you have to turn to other methods/platforms/technologies to perform tasks that were meant to be completed in this platform?
    - Was the experience by other users similar or different?
13. How would you describe the complexity of these platforms?
    - How well did they function overall?
14. How likely or often are you to use this platform in the future?
    - What tasks would you continue to perform using [platform]?
    - Why would you discontinue to use [platform]?
    - Could that be corrected?
15. How satisfied are you with the product price?
