# Supplementary material for: Best Practices for Virtual Engagement of Patient-Centered Outcomes Research Teams During and After the COVID-19 Pandemic: Qualitative Study
Source: J Particip Med. 2021 Mar 11;13(1):e24966. doi: 10.2196/24966 (PMC7954110; doi:10.2196/24966)
Supplement: Multimedia Appendix 2 [file jopm_v13i1e24966_app2.docx]

## **Multimedia Appendix 2.** Benefits and Challenges of Specific Online Platforms

| **VIDEO CONFERENCING PLATFORMS**  *Platforms that allow teams to communicate over video* | | | | | | | | | | | | | | | | | | | | |
| --- | --- | --- | --- | --- | --- | --- | --- | --- | --- | --- | --- | --- | --- | --- | --- | --- | --- | --- | --- | --- |
| **Attributes** | **Zoom** | | | **BlueJeans** | | **WebEx** | | **Google Meet** | | **Facetime** | | | **WhatsApp** | | | **Skype** | | | **GoToMeeting** |  |
| **Cost to host** | Different levels: free (limited), personal, institutional;  Free to join a meeting | | | Institution typically pays | | Starter, Plus, Business | | Free with Gmail account (during pandemic), prior for enterprise | | Free, limited to people with apple devices | | | Free | | | Free (limited), business (fee per user) | | | Different levels: free trial, professional, business, enterprise |  |
| **Bandwidth** | Works with low bandwidth | | | Requires stable internet | | Works with low bandwidth | | Requires stable internet | | Works with low bandwidth | | | Works with Low bandwidth; also works over cell signal | | | Requires stable internet; also works over cell signal | | | Requires stable internet |  |
| **Security** ^[[1]](#footnote-2)^ | Zoom Healthcare HIPAA-  Compliant; others not | | | HIPAA-  Compliant | | HIPAA-  Compliant | | HIPAA-compliant; Google Hangouts not HIPAA compliant | | End-to end encryption, but not HIPAA compliant | | | End-to end encryption, but not HIPAA compliant | | | Free version not HIPAA compliant; Business E3 and E5 packages are | | | HIPAA- compliant |  |
| **Attributes** | **Zoom** | | | **BlueJeans** | | **WebEx** | | **Google Meet** | | **Facetime** | | | **WhatsApp** | | | **Skype** | | | **GoToMeeting** |  |
| **Privacy** | Optional meeting password; waiting room; disable screen share | | | Optional meeting password | | Encrypted; offers user authentication to optimize security | | Only emails included in invite allowed into meeting without approval; others allowed with approval | | Must have number in phone contacts | | | Requires participant phone number | | | Must have number in phone contacts | | | Uses “LogMeIn” protocols; Meeting Lock and Password Protected Meetings |  |
| **Accommodation/ disability support** | Integrates with captioning software | | | Automatic Captioning capabilities | | Closed captioning | | Live translation and captioning in English | | Live translation into English | | | Options for mobile device | | | Live translation | | | Automatic captioning capabilities |  |
| **Integration** | Integrates with Outlook, Google drive, Dropbox, file sharing in chat | | | Integrates with Outlook, Google calendar, Microsoft 365, file share in chat | | Integrates with Microsoft | | Integrates with all Google products | | Integrates with Apple products | | | Integrates with Facebook Messenger, Instagram | | | Integrates with Outlook, Slack, Microsoft Systems | | | Integrates with Outlook and Google Calendar |  |
| **Other Benefits** |  | | |  | | Multiple users can control screen share | | No plug ins or desktop app required; ability to share tab not just window in screen share | |  | | |  | | |  | | | Multiple users can control share screen |  |
| **Attributes** | **Zoom** | | | **BlueJeans** | | **WebEx** | | **Google Meet** | | **Facetime** | | | **WhatsApp** | | | **Skype** | | | **GoToMeeting** |  |
| **Other Challenges** | Some features not available on web version | | | Requests meeting rating each time; Some features not available on web version | | Works easiest with Internet Explorer, note taking application | | Difficult to use for non-Gmail users; no whiteboard or poll/survey options | | Only available for Apple products; Apple collects metadata | | | Limited to 4 people per video-chat | | | Limited options/ features | | | Requires newer equipment |  |
| **DOCUMENT STORAGE PLATFORMS**  *Platforms that allow teams to share and collaborate on documents* | | | | | | | | | | | | | | | | | | | | |
| **Attribute** | | **Dropbox** | | | **Google Drive** | | **Box** | | | | **Microsoft OneDrive** | | | | **Email** | | | | **Egynte** |  |
| **Collaboration Type** | | File share | | | Collaborative editing | | File share | | | | Collaborative editing | | | | File share | | | | File Share |  |
| **Integration** | | Any desktop word processor | | | G-Suite, Slack | | Any desktop word processor, G-suite, Slack | | | | Open API^^[[2]](#footnote-3)^^, can be added to any app | | | | Any desktop word processor | | | | Microsoft word |  |
| **Editing type** | | Asynchronous | | | Both synchronous and asynchronous | | Both synchronous and asynchronous | | | | Both synchronous and asynchronous | | | | Asynchronous | | | | Asynchronous |  |
| **Cost** | | Free up to 2GB data | | | Free | | Free and paid options | | | | Free up to 5GB | | | | No free option; Subscription based | | | | No free option; Subscription based |  |
| **Attribute** | | **Dropbox** | | | **Google Drive** | | **Box** | | | | **Microsoft OneDrive** | | | | **Email** | | | | **Egynte** |  |
| **Other Benefits** | | Easily accessible anywhere on any device; sync feature; system integration | | | Easy to edit files; Easy to locate documents, | | View project Timelines; collaborate in real time | | | | Collaboration via Microsoft 365; easy to use | | | | Shared mailbox; Great for small teams | | | | Share files outside of organization; ease of use and loading |  |
| **Other Challenges** | | Cannot edit in real time; limited search function | | | Limited to Gmail; some document access requires permission | | Lacks integration with other solutions; difficult document creation | | | | Slow interface; difficulty syncing and document management | | | | Expense of licensing; following email chains | | | | Syncing documents and moving documents difficult; cannot share in real time |  |
| **SCHEDULING PLATFORMS**  *Platforms that allow groups to coordinate meeting times* | | | | | | | | | | | | | | | | | | | | |
| **Attribute** | | | **Doodle** | | | **Calendly** | | | | | | **Google Calendar** | | | | | **When2Meet** | | | |
| **Use-case** | | | List of specific times to meet | | | Scheduling a meeting with a specific person by looking at their personal calendar availability | | | | | | Individual event scheduling or scheduling with a single person’s calendar | | | | | Overlapping time blocks to see all participants’ availability | | | |
| **User-friendliness** | | | Easy to use for specific dates and times for poll creation | | | Easy to mark time availability | | | | | | Easy to use to schedule an event and send invites | | | | | Easy to use for time availability; can be confusing to set up | | | |
| **Integration** | | | Google, Office 365/ Outlook, Zoom, Slack | | | Outlook, Google Calendar and Microsoft Office 365 | | | | | | Use applications or add-ons for integration with other platforms | | | | | None | | | |
| **Other Benefits** | | | Easy to set up and send either email or link | | | Offers location and personalized invitations; sends reminder email | | | | | | Meeting alerts, syncing with Gmail | | | | | Provides availability surveys | | | |
| **Attribute** | | | **Doodle** | | | **Calendly** | | | | | | **Google Calendar** | | | | | **When2Meet** | | | |
| **Other Challenges** | | | Works best with few options of times to meet; harder to use on mobile devices; free plan has limited options | | | Lacks integration options | | | | | | Lacks integration options | | | | |  | | | |
| **INSTANT MESSAGING AND COMMUNICATION PLATFORMS**  *Platforms that allow teams to text or exchange messages in real time* | | | | | | | | | | | | | | | | | | | | |
| **Attribute** | | | **Email** | | | **Slack** | | | **WhatsApp** | | | | | **SMS** | | | | **Skype** | | |
| **Cost** | | | Free | | | Free up to 10,000 messages, then messages disappear | | | Free | | | | | Unless using work phones, cost may fall to team members | | | | Cost dependent on number of users | | |
| **Integration** | | | Can integrate with calendars, some with video conferencing platforms | | | Can integrate with various other platforms (e.g., Google Drive) | | | Can share links, documents, photos | | | | | Can share links, photos, documents | | | | Outlook Email | | |
| **Searchable Chat Record** | | | Yes | | | Yes (Can search within channels or by person) | | | Yes | | | | | Yes | | | | If integrated with Outlook Email | | |
| **Security** | | | Depends on the email platform | | | Password protected, can limit access to specific channels | | | End-to-end encryption | | | | | Not encrypted | | | | Encryption | | |
| **Accommodation/ disability support** | | | Voice message available depending on platform | | |  | | | Voice message | | | | | Voice message | | | | Voice message | | |
| **Attribute** | | | **Email** | | | **Slack** | | | **WhatsApp** | | | | | **SMS** | | | | **Skype** | | |
| **Other Benefits** | | | Can be used all times of the day without disturbing people in different time zones. | | | Works on computers and all smart phones through app | | | Similar to texting; phone number required; group messaging with all seeing same message(s) | | | | |  | | | |  | | |
| **Other Challenges** | | | Long email chains - difficulty in following conversation | | | Numerous notifications | | | Multiple person format means long message chains | | | | |  | | | |  | | |

1. Department of Health and Human Services. Notification of Enforcement Discretion for Telehealth Remote Communications During the COVID-19 Nationwide Public Health Emergency. Office of Civil Rights Headquarters. <https://www.hhs.gov/hipaa/for-professionals/special-topics/emergency-preparedness/notification-enforcement-discretion-telehealth/index.html> Published 2020. Accessed October 11, 2020. [Webcite not available] [↑](#footnote-ref-2)
2. ^b.^ Application program interface (API) means the platform is made publicly available to web developers. Rouse M, McKenzie C. Open API (public API). Techtarget Network. App Architecture Web site. <https://searchapparchitecture.techtarget.com/definition/open-API-public-API>. Published 2019. Accessed October 11, 2020. [↑](#footnote-ref-3)
